# Supplementary material for: Dynamic Change of Aroma Components in Chimonanthus praecox Flower Scented Teas During Absorption and Storage
Source: Foods. 2025 May 11;14(10):1696. doi: 10.3390/foods14101696 (PMC12111064; doi:10.3390/foods14101696)
Supplement: Supplementary file 1 [file foods-14-01696-s001.zip › foods-3588141-supplementary.pdf]

Supporting Information

for

Dynamic Change of Aroma Components in *Chimonanthus praecox* Flower Scented Teas During Absorption and Storage

Xiongyuan Si [1,†], Hao Zuo [2,†], Penghui Li [3], Ye Tan [1], Mangmang Tan [3], Zhihui Chen [4], Changsong Chen [4], Taolin Chen [5], Zhonghua Liu [2], Jian Zhao [2,*]

[1] Biotechnology Center, Anhui Agricultural University, Hefei 230036, China; xysi@ahau.edu.cn (X.S.); tanye1213@163.com (Y.T.)
[2] Key Laboratory of Tea Science of Ministry of Education, College of Horticulture, Hunan Agricultural University, Changsha 410128, China; zuohao0116@163.com (H.Z.); zhonghua-liu@hunau.edu.cn (Z.L.)
[3] State Key Laboratory of Tea Plant Biology and Utilization, Anhui Agricultural University, Hefei 230036, China; lphui2012@126.com (P.L.); tanmang07@163.com (M.T.)
[4] Tea Research Institute, Fujian Academy of Agricultural Sciences, Fuzhou 350013, China; chenzhihui75@sina.com (Z.C.); ccs6536597@163.com (C.C.)
[5] College of Tea Science, Guizhou University, Guiyang 550025, China; tlchen@gzu.edu.cn
* Correspondence: zhaojian@hunau.edu.cn; Tel.: +86-18674047685
† These authors contributed equally to this work.

*** To whom correspondence should be addressed:**

Jian Zhao, Ph.D.

Professor

Key Laboratory of Tea Science of Ministry of Education,

College of Horticulture, Hunan Agricultural University, Changsha, 410128, China.

Phone: +86 18674047685

Email: jzhao2@qq.com; zhaojian@hunau.edu.cn

Profile URL: https://www.researchgate.net/profile/Jian_Zhao16

Profile: https://orcid.org/0000-0002-4416-7334

UPL: https://www.ncbi.nlm.nih.gov/myncbi/1HCl6YPtG78ke/bibliography/public/

Table S1. Identification of aroma of *Chimonanthus praecox* flower

Name	Calculated RI (Literature RI)	CAS	Precentage of content-1	Precentage of content-2	Precentage of content-3	Precentage of content(average)	SD
**Alcohols**							
Phenylmethanol	1057(1052)	100-51-6	2.7156	3.2516	2.1827	2.7166	0.3566
Linalool oxide I	1094(1090)	5989-33-3	0.2350	0.2101	0.2630	0.2360	0.0180
Linalool oxide II	1109(1099)	34995-77-2	0.3977	0.3688	0.4295	0.3987	0.0206
Linalool	1129(1114)	78-70-6	48.2973	50.2414	46.3622	48.3003	1.2941
4-Terpineol	1211(1192)	562-74-3	0.0300	0.0347	0.0284	0.0310	0.0024
3-Phenylpropanol	1262(1243)	122-97-4	0.0239	0.0223	0.0295	0.0252	0.0028
Cinnamyl alcohol	1341(1330)	104-54-1	0.2197	0.2113	0.2712	0.2341	0.0247
**Aldehydes**							
Phenylmethanal	983(982)	100-52-7	0.1023	0.1258	0.1118	0.1133	0.0083
(E)-Cinnamaldehyde	1306(1287)	14371-10-9	0.1045	0.1059	0.1260	0.1121	0.0093
**Alkanes**							
2,4-Hexadiene	640(650)	592-46-1	0.0652	0.0797	0.0888	0.0779	0.0084
Toluene	772(769)	108-88-3	0.0172	0.0186	0.0189	0.0182	0.0007
Octane	807(800)	111-65-9	0.0204	0.0191	0.0213	0.0202	0.0008
Phenylethane	874(865)	100-41-4	0.0214	0.0243	0.0236	0.0231	0.0011
p-Xylene	883(876)	106-42-3	0.0283	0.0325	0.0311	0.0307	0.0016
Styrene	907(897)	100-42-5	0.4638	0.3941	0.5365	0.4648	0.0478
α-Thujene	943(938)	2867-05-2	0.6695	0.6466	0.6953	0.6705	0.0166
α-Pinene	952(946)	80-56-8	0.0438	0.0529	0.0478	0.0482	0.0032
Sabinene	993(984)	3387-41-5	1.0718	1.3705	0.9761	1.1394	0.1540
β-Pinene	1000(995)	127-91-3	0.0312	0.0318	0.0336	0.0322	0.0009

β-Myrcene	1006(1001)	123-35-3	0.7419	0.6783	0.8085	0.7429	0.0437
α-Phellandrene	1028(1014)	99-83-2	0.3649	0.4218	0.3811	0.3893	0.0217
α-Terpinene	1039(1026)	99-86-5	0.2416	0.2818	0.2345	0.2526	0.0194
o-Cymene	1047(1038)	527-84-4	0.1966	0.2141	0.1821	0.1976	0.0110
Limonene	1052(1040)	138-86-3	0.2332	0.2348	0.2347	0.2342	0.0007
β-Ocimene	1054(1046)	13877-91-3	0.4578	0.4436	0.3751	0.4255	0.0336
(E)-β-Ocimene	1065(1054)	3779-61-1	1.0942	0.9412	0.8856	0.9737	0.0804
γ-Terpinene	1081(1073)	99-85-4	0.5688	0.6795	0.5612	0.6031	0.0509
Allo-Ocimene	1150(1139)	673-84-7	0.1398	0.1240	0.1587	0.1408	0.0119
1,3,8-p-Menthatriene	1154(NA)	18368-95-1	0.0866	0.0895	0.0867	0.0876	0.0012
δ-EIemene	1369(1361)	20307-84-0	0.0179	0.0198	0.0190	0.0189	0.0007
Ylangene	1407(1391)	14912-44-8	0.0193	0.0221	0.0225	0.0213	0.0013
Copaene	1414(1405)	3856-25-5	0.0314	0.0315	0.0304	0.0311	0.0005
Longifolene	1425(1408)	475-20-7	0.0420	0.0453	0.0417	0.0430	0.0015
(Z)-Muurola-3,5-diene	1432(1442)	NA	0.0133	0.0147	0.0154	0.0145	0.0008
Caryophyllene	1463(1450)	87-44-5	0.0438	0.0514	0.0493	0.0482	0.0029
γ-Elemene	1466(1455)	3242-08-8	0.0328	0.0351	0.0335	0.0338	0.0009
Aristolene	1480(1486)	6831-16-9	0.0834	0.0837	0.0761	0.0810	0.0033
Isoledene	1490(1482)	95910-36-4	0.0155	0.0177	0.0183	0.0172	0.0011
α-Humulene	1499(1489)	6753-98-6	0.0209	0.0192	0.0216	0.0206	0.0009
Bicyclosesquiphellandrene	1504(1491)	54324-03-7	0.0165	0.0177	0.0182	0.0175	0.0006
γ-Muurolene	1514(1496)	30021-74-0	0.0231	0.0248	0.0243	0.0241	0.0006
δ-Cadinene	1519(1518)	483-76-1	0.0172	0.0187	0.0207	0.0188	0.0012
β-Guaiene	1525(1522)	88-84-6	0.0234	0.0263	0.0235	0.0244	0.0013
α-Muurolene	1538(1534)	10208-80-7	0.0160	0.0172	0.0178	0.0170	0.0007
α-Selinene	1541(1534)	473-13-2	0.0190	0.0205	0.0204	0.0200	0.0006

β-Cadinene	1550(1530)	523-47-7	0.0192	0.0199	0.0215	0.0202	0.0009
γ-Cadinene	1557(1543)	39029-41-9	0.0335	0.0352	0.0348	0.0345	0.0007
(E)-Calamenene	1563(1561)	73209-42-4	0.0266	0.0277	0.0284	0.0276	0.0006
**Esters**							
Pentyl acetate	925(915)	628-63-7	0.0161	0.0166	0.0177	0.0168	0.0006
Benzyl formate	1100(1079)	104-57-4	0.0196	0.0211	0.0212	0.0206	0.0007
Phenylmethyl acetate	1190(1180)	140-11-4	26.1991	23.6614	27.0553	25.6386	1.3181
Methyl salicylate	1224(1222)	119-36-8	10.1801	10.0283	11.8350	10.6811	0.7692
Methyl anthranilate	1376(1363)	134-20-3	0.0642	0.0394	0.0919	0.0652	0.0178
γ-Phenylpropyl acetate	1398(1380)	122-72-5	0.0528	0.0482	0.0737	0.0582	0.0103
(Z)-Cinnamyl acetate	1417(1409)	77134-01-1	0.0293	0.0308	0.0308	0.0303	0.0007
(E)-Cinnamyl acetate	1477(1455)	103-54-8	0.4031	0.3493	0.3599	0.3708	0.0216
**Ketones**							
Pulegone	958(NA)	15932-80-6	0.0458	0.0414	0.0603	0.0492	0.0074
1-Phenylethanone	1090(1079)	98-86-2	0.0182	0.0213	0.0211	0.0202	0.0013
**Nitrogen compounds**							
Indole	1326(1329)	120-72-9	2.8703	2.9042	2.8394	2.8713	0.0220
**Phenols**							
Eugenol	1383(1373)	97-53-0	0.8866	0.9228	0.8534	0.8876	0.0235
(E)-Isoeugenol	1437(1445)	5932-68-3	0.0157	0.0164	0.0179	0.0167	0.0008

Table S2. Identification of aroma of Large-leaf Yellow Tea (LLYT)

Name	Calculated RI (Literature RI)	CAS	Precentage of content-1	Precentage of content-2	Precentage of content-3	Precentage of content(average)	SD
**Alcohols**							
(E)-3-Hexenol	868(862)	928-97-2	0.1859	0.2361	0.2079	0.2100	0.0174
Hexanol	880(871)	111-27-3	0.0582	0.0566	0.0640	0.0596	0.0030
3-Octenol	997(985)	3391-86-4	0.3416	0.3182	0.3540	0.3379	0.0132
Linalool oxide I	1094(1090)	5989-33-3	2.5802	3.3654	2.6535	2.8664	0.3327
Linalool oxide II	1111(1099)	34995-77-2	3.1709	3.4393	3.3440	3.3180	0.0981
Linalool	1122(1114)	78-70-6	2.4138	2.6464	2.5252	2.5285	0.0786
Hotrienol	1126(1109)	20053-88-7	5.1038	6.4294	4.4533	5.3288	0.7337
Geraniol	1275(1259)	106-25-2	0.6746	0.6964	0.6401	0.6704	0.0202
**Aldehydes**							
Isobutanal	NA(540)	78-84-2	0.1145	0.0989	0.0940	0.1024	0.0080
3-Methylbutanal	656(658)	590-86-3	0.2066	0.2138	0.1971	0.2059	0.0058
2-Methylbutanal	665(659)	96-17-3	0.7664	0.5878	0.6816	0.6786	0.0605
Hexanal	809(809)	66-25-1	0.1355	0.1441	0.1371	0.1389	0.0035
3-Furaldehyde	842(842)	498-60-2	2.9970	2.1467	2.6693	2.6043	0.3051
5-Methylfurfural	978(978)	620-02-0	1.5347	0.8678	1.1765	1.1930	0.2278
Phenylmethanal	983(982)	100-52-7	2.4992	2.0200	1.9941	2.1711	0.2187
(E,E)-2,4-Heptadienal	1031(1020)	4313-03-5	0.1821	0.2845	0.2466	0.2377	0.0371
Phenylethanal	1068(1058)	122-78-1	1.2067	1.2073	1.0126	1.1422	0.0864
Decanal	1230(1231)	112-31-2	0.9439	0.8949	0.9860	0.9416	0.0312
β-Cyclocitral	1239(1227)	432-25-7	0.4561	0.5394	0.5711	0.5222	0.0441
**Alkanes**							

Toluene	773(769)	108-88-3	0.9273	1.0090	1.0342	0.9902	0.0419
1,3-Xylene	883(869)	108-38-3	1.0566	1.3387	1.0640	1.1531	0.1237
2,6-Dimethyl-1,5-heptadiene	894(882)	6709-39-3	0.1224	0.1327	0.1334	0.1295	0.0047
Styrene	909(897)	100-42-5	0.3747	0.4884	0.3399	0.4010	0.0583
β-Myrcene	1007(1001)	123-35-3	1.1612	0.8599	1.0058	1.0090	0.1015
α-Terpinene	1039(1026)	99-86-5	0.3633	0.2852	0.3496	0.3327	0.0317
o-Cymene	1047(1038)	527-84-4	0.3733	0.3985	0.4125	0.3948	0.0143
Limonene	1052(1040)	138-86-3	1.1715	0.8660	1.0164	1.0180	0.1024
β-Ocimene	1054(1046)	13877-91-3	0.6266	0.5685	0.6417	0.6123	0.0291
3,4-Dehydroionene	1392(1398)	30364-38-6	1.3482	1.0128	1.1532	1.1714	0.1179
Neophytadiene	1869(1844)	504-96-1	0.0841	0.1042	0.1002	0.0962	0.0081
**Esters**							
Methyl 1,5-dimethyl-2-pyrrolecarboxylate	1183(NA)	73476-31-0	0.5056	0.5626	0.5818	0.5500	0.0296
Methyl phenylacetate	1200(1180)	101-41-7	1.0996	1.0016	1.2847	1.1286	0.1041
Methyl salicylate	1223(1222)	119-36-8	5.3983	6.1568	5.1881	5.5811	0.3838
Dihydroactinidiolide	1562(1539)	17092-92-1	0.1534	0.2027	0.1738	0.1766	0.0174
**Ketones**							
Propylacetone	796(791)	591-78-6	0.0313	0.0251	0.0252	0.0272	0.0027
2-Heptanone	901(891)	110-43-0	1.2974	1.2833	1.1648	1.2485	0.0558
Prenylacetone	1002(995)	110-93-0	1.1521	1.5161	1.1201	1.2627	0.1689
1-(1H-pyrrol-2-yl) ethanone	1086(1075)	1072-83-9	0.4427	0.4565	0.4191	0.4394	0.0136
2-Decanone	1214(1194)	693-54-9	0.2804	0.3404	0.3688	0.3299	0.0330
Jasmone	1427(1415)	488-10-8	0.1731	0.1625	0.1315	0.1557	0.0161
α-Ionone	1456(1438)	127-41-3	0.1713	0.2368	0.1661	0.1914	0.0303
Dehydro-β-ionone	1462(1460)	1203-08-3	0.1441	0.0923	0.1393	0.1252	0.0220

Geranylaceton	1476(1461)	3796-70-1	0.2234	0.1456	0.2000	0.1896	0.0294
2,6-Di-tert-butylquinone	1487(1472)	719-22-2	0.0718	0.0835	0.0828	0.0793	0.0050
(E)-β-Ionone	1500(1490)	79-77-6	0.4756	0.4025	0.3613	0.4132	0.0417
**Nitrogen compounds**							
1-Methylpyrrole	736(743)	96-54-8	0.1781	0.1528	0.2250	0.1853	0.0265
2,5-Dimethylpyrazine	929(923)	123-32-0	8.0773	5.5635	7.4180	7.0196	0.9708
1,2,5-Trimethylpyrrole	936(934)	930-87-0	3.0630	2.3283	2.6255	2.6723	0.2605
1-Butyl-1H-pyrrole	963(980)	589-33-3	0.1424	0.1745	0.1417	0.1529	0.0144
2-Ethyl-3-methylpyrazine	1021(1015)	15707-23-0	7.9774	8.3490	7.8003	8.0422	0.2045
2-Ethyl-5-methylpyrazine	1022(1022)	13360-64-0	7.3780	7.8664	8.9735	8.0726	0.6006
3-Ethyl-2,5-dimethylpyrazine	1096(1091)	13360-65-1	7.4726	8.6443	8.9018	8.3395	0.5780
2,6-Diethylpyrazine	1103(1085)	13067-27-1	1.0316	1.1607	1.1697	1.1207	0.0594
2,5-Diethylpyrazine	1114(1095)	13238-84-1	0.8381	0.6849	0.6612	0.7281	0.0734
p-Anisidine	1136(NA)	104-94-9	8.3168	8.1761	8.0202	8.1711	0.1005
α-Cyanotoluene	1164(1144)	140-29-4	0.4127	0.4333	0.4063	0.4174	0.0106
2-Allyl-6-methylpyrazine	1170(1170)	55138-64-2	0.2461	0.2090	0.2448	0.2333	0.0162
2,3-Diethyl-5-methylpyrazine	1173(1165)	18138-04-0	0.6695	0.9002	0.7957	0.7884	0.0793
2-Methyl-3,5-diethylpyrazine	1177(1167)	18138-05-1	2.2480	2.8363	3.1735	2.7526	0.3364
3,5-Dimethyl-2-propylpyrazine	1179(1152)	32350-16-6	0.0255	0.0321	0.0307	0.0294	0.0026
2,5-Dimethyl-3-isopropylpyrazine	1187(NA)	40790-20-3	0.6114	0.6926	0.5979	0.6340	0.0391
1-Furfurylpyrrole	1204(1197)	1438-94-4	1.6129	1.2743	1.3164	1.4012	0.1411
Indole	1328(1329)	120-72-9	0.6993	0.5188	0.6578	0.6253	0.0710
2,5-Dimethyl-3-isopentylpyrazine	1339(1323)	18433-98-2	0.2460	0.3152	0.3571	0.3061	0.0401
1,7-Trimethylene-2,3-dimethylindole	1659(NA)	5825-43-4	0.0753	0.0999	0.0847	0.0866	0.0088
**Oxygen heterocyclic**							
3-Methylfuran	611(614)	930-27-8	0.5584	0.4412	0.4583	0.4860	0.0483

2,5-dimethylfuran	705(707)	625-86-5	0.0350	0.0431	0.0415	0.0399	0.0032
2-Methoxyfuran	806(NA)	25414-22-6	0.2857	0.2413	0.2256	0.2509	0.0232
2,3-Dihydrobenzofuran	1230(1226)	496-16-2	0.0620	0.0615	0.0424	0.0553	0.0086
**Phenols**							
4-Amino-2,6-dimethylphenol	1207(NA)	15980-22-0	2.9360	2.8758	2.9636	2.9251	0.0329

Table S3. Identification of aroma of Dianhong jinzhen Tea (DHJZ)

Name	Calculated RI (Literature RI)	CAS	Precentage of content-1	Precentage of content-2	Precentage of content-3	Precentage of content(average)	SD
**Alcohols**							
2-Heptanol	913(905)	543-49-7	0.2036	0.1372	0.1883	0.1764	0.0261
3-Octenol	997(985)	3391-86-4	0.1064	0.0889	0.0949	0.0968	0.0064
Phenylmethanol	1057(1052)	100-51-6	0.6938	0.6099	0.6306	0.6448	0.0327
Linalool oxide I	1094(1090)	5989-33-3	4.9601	4.5541	4.8711	4.7951	0.1606
Linalool oxide II	1111(1099)	34995-77-2	14.2174	15.3673	15.2259	14.9369	0.4796
Linalool	1122(1114)	78-70-6	24.8699	32.6430	27.6043	28.3724	2.8471
Hotrienol	1126(1109)	20053-88-7	3.0086	3.2836	4.0290	3.4404	0.3924
Phenylethanol	1139(1129)	60-12-8	11.4256	8.3187	11.9082	10.5508	1.4881
Linalool oxide IV	1201(1183)	39028-58-5	4.6362	4.0148	5.2311	4.6274	0.4084
4-Terpineol	1211(1192)	562-74-3	0.0954	0.1031	0.0963	0.0983	0.0032
α-Terpineol	1225(1192)	10482-56-1	1.7955	1.4879	1.4313	1.5716	0.1493
(E)-p-2,8-Mentha-dienol	1251(1221)	NA	0.2823	0.2157	0.3257	0.2746	0.0392
Isogeraniol	1265(1248)	5944-20-7	0.2630	0.2029	0.2305	0.2321	0.0206
Geraniol	1275(1259)	106-25-2	1.4935	1.4159	1.7633	1.5576	0.1372
Nerolidol	1593(1585)	142-50-7	0.2051	0.1610	0.1453	0.1705	0.0231
**Aldehydes**							
3-Methyl-butanal	656(658)	590-86-3	0.4458	0.4351	0.3939	0.4249	0.0207
2-Methyl-butanal	665(659)	96-17-3	1.3361	1.3129	0.9612	1.2034	0.1615
2-Methylpentanal	765(760)	123-15-9	0.0969	0.0776	0.0747	0.0831	0.0092
Hexanal	809(809)	66-25-1	0.2522	0.2034	0.2600	0.2385	0.0234
3-Furaldehyde	842(842)	498-60-2	0.9458	0.9248	0.7636	0.8780	0.0763

(E)-2-Hexanal	864(855)	6728-26-3	0.3934	0.3399	0.3636	0.3656	0.0185
Heptanal	915(905)	111-71-7	0.2865	0.2423	0.2545	0.2611	0.0169
Phenylmethanal	983(982)	100-52-7	1.8208	1.6730	1.5804	1.6914	0.0862
Octanal	1022(1013)	124-13-0	0.1596	0.1591	0.1541	0.1576	0.0023
Phenylethanal	1068(1058)	122-78-1	6.0072	4.3163	4.5251	4.9495	0.7051
Decanal	1230(1231)	112-31-2	0.6272	0.4089	0.4946	0.5102	0.0780
Citral	1294(1276)	5392-40-5	0.0946	0.0765	0.0659	0.0790	0.0104
Undecanal	1333(1313)	112-44-7	0.1218	0.0892	0.1011	0.1040	0.0119
Dodecanal	1437(1419)	112-54-9	0.2783	0.2715	0.3064	0.2854	0.0140
Tetradecanal	1645(1623)	124-25-4	0.1236	0.1043	0.1026	0.1102	0.0090
**Alkanes**							
Toluene	773(769)	108-88-3	0.2487	0.2038	0.1938	0.2155	0.0222
Styrene	909(897)	100-42-5	0.2573	0.2094	0.2722	0.2463	0.0246
β-Myrcene	1007(1001)	123-35-3	0.6430	0.7142	0.6588	0.6720	0.0281
α-Terpinene	1039(1026)	99-86-5	0.0525	0.0387	0.0496	0.0469	0.0055
o-Cymene	1047(1038)	527-84-4	0.9219	0.7233	0.6524	0.7659	0.1040
Limonene	1052(1040)	138-86-3	3.4691	3.2179	3.4756	3.3875	0.1131
β-Ocimene	1054(1046)	13877-91-3	0.2983	0.2542	0.2660	0.2728	0.0170
**Esters**							
Ethyl acetate	620(614)	141-78-6	0.3631	0.3498	0.3447	0.3525	0.0071
Propyl acetate	711(712)	109-60-4	0.6569	0.6756	0.5054	0.6126	0.0715
Phenylmethyl acetate	1187(1165)	140-11-4	0.2370	0.1949	0.2291	0.2203	0.0169
Methyl salicylate	1223(1222)	119-36-8	8.4555	7.7756	6.5183	7.5831	0.7099
Methyl 2,4,6-trimethylundecanoate	1519(1535)	55955-76-5	0.1149	0.1024	0.1029	0.1067	0.0054
Txib	1617(1588)	6846-50-0	0.9345	0.6937	0.7804	0.8029	0.0878

**Ketones**							
2-Heptanone	901(892)	110-43-0	0.2290	0.1839	0.2140	0.2090	0.0167
Prenylacetone	1002(995)	110-93-0	0.2953	0.2100	0.2472	0.2508	0.0297
β-Damascenone	1413(1395)	23726-93-4	0.1202	0.0892	0.1086	0.1060	0.0112
Geranylaceton	1476(1461)	3796-70-1	0.2102	0.1440	0.2154	0.1899	0.0306
2,6-Di-tert-butylquinone	1487(1472)	719-22-2	0.4559	0.2910	0.2820	0.3430	0.0753
(E)-β-Ionone	1500(1490)	79-77-6	0.1838	0.1443	0.1673	0.1651	0.0139
**Oxygen heterocyclic**							
2-Methoxyfuran	806(NA)	25414-22-6	0.2652	0.2219	0.2519	0.2463	0.0163
**Phenols**							
4-Amino-2,6-dimethylphenol	1207(NA)	15980-22-0	0.2529	0.2440	0.2076	0.2348	0.0182
Eugenol	1384(1373)	97-53-0	0.0888	0.0790	0.0797	0.0825	0.0042

Table S4. Identification of aroma of Huangshan Maofeng Tea (HSMF)

Name	Calculated RI (Literature RI)	CAS	Precentage of content-1	Precentage of content-2	Precentage of content-3	Precentage of content(average)	SD
**Alcohols**							
Penten-3-ol	686(684)	616-25-1	0.3475	0.3327	0.3402	0.3401	0.0049
Pentanol	773(768)	71-41-0	1.5349	1.4999	1.4182	1.4843	0.0441
(E)-3-Hexenol	868(862)	928-97-2	4.2061	3.8949	3.4599	3.8536	0.2625
Hexanol	880(871)	111-27-3	0.7100	0.5931	0.8479	0.7170	0.0873
3-Octenol	997(985)	3391-86-4	0.5983	0.5064	0.5456	0.5501	0.0321
Phenylmethanol	1057(1052)	100-51-6	0.3113	0.4039	0.4132	0.3761	0.0432
Linalool oxide I	1094(1090)	5989-33-3	3.8018	3.9274	2.9247	3.5513	0.4177
Linalool oxide II	1111(1099)	34995-77-2	6.6792	7.2036	5.3634	6.4154	0.7013
Linalool	1122(1114)	78-70-6	12.2703	9.1493	10.7275	10.7157	1.0443
Phenylethanol	1139(1129)	60-12-8	1.4286	1.6917	1.5590	1.5598	0.0879
Linalool oxide IV	1203(1183)	39028-58-5	2.3256	2.3517	2.3724	2.3499	0.0162
Geraniol	1275(1259)	106-25-2	12.3094	12.9092	12.2519	12.4902	0.2794
(E)-Geraniol	1297(1295)	106-24-1	0.2109	0.1921	0.2016	0.2015	0.0063
Nerolidol	1593(1585)	142-50-7	0.1843	0.2392	0.1912	0.2049	0.0228
**Aldehydes**							
3-Methyl-butanal	656(658)	590-86-3	0.2489	0.2810	0.3047	0.2782	0.0195
2-Methyl-butanal	665(659)	96-17-3	0.4783	0.4399	0.5228	0.4803	0.0283
Pentanal	702(703)	110-62-3	1.0363	0.9830	0.8988	0.9727	0.0493
Hexanal	809(809)	66-25-1	5.9842	5.4326	6.7169	6.0446	0.4483
2-Methyl-2-pentenal	840(828)	623-36-9	0.1724	0.1244	0.1415	0.1461	0.0176
Heptanal	915(905)	111-71-7	1.5096	1.6043	1.4631	1.5257	0.0524

Phenylmethanal	983(982)	100-52-7	4.0253	4.3092	4.5471	4.2939	0.1790
Octanal	1022(1013)	124-13-0	1.0466	0.9880	1.1373	1.0573	0.0534
Phenylethanal	1068(1058)	122-78-1	0.5577	0.6670	0.7569	0.6605	0.0686
Nonanal	1128(1112)	124-19-6	1.9687	2.0206	2.6769	2.2221	0.3032
Decanal	1230(1231)	112-31-2	1.0516	0.8562	1.2778	1.0619	0.1440
Citral	1294(1276)	5392-40-5	0.2947	0.3033	0.2954	0.2978	0.0037
Undecanal	1333(1313)	112-44-7	0.0882	0.1163	0.1022	0.1022	0.0094
Dodecanal	1437(1419)	112-54-9	0.1334	0.0746	0.1044	0.1041	0.0197
**Alkanes**							
Toluene	773(769)	108-88-3	5.4957	6.9177	6.2002	6.2046	0.4755
p-Xylene	884(876)	106-42-3	0.4068	0.4115	0.3058	0.3747	0.0459
Styrene	909(897)	100-42-5	0.5874	0.7014	0.6641	0.6509	0.0424
α-Pinene	952(946)	80-56-8	2.3054	2.7686	3.1511	2.7417	0.2909
β-Myrcene	1007(1001)	123-35-3	0.6481	0.8631	1.0496	0.8536	0.1370
α-Phellandrene	1031(1014)	99-83-2	1.0134	1.4026	1.4822	1.2994	0.1907
o-Cymene	1047(1038)	527-84-4	0.9924	0.7892	1.2245	1.0020	0.1483
Limonene	1052(1040)	138-86-3	2.7057	2.2723	3.2191	2.7324	0.3245
β-Ocimene	1054(1046)	13877-91-3	0.2872	0.3754	0.3309	0.3312	0.0295
Copaene	1381(1377)	3856-25-5	0.1710	0.2413	0.2225	0.2116	0.0271
α-Cedrene	1461(1438)	469-61-4	1.3144	1.3354	1.3347	1.3282	0.0092
γ-Muurolene	1471(1496)	30021-74-0	0.1269	0.1900	0.2479	0.1883	0.0409
Thujopsene	1477(1450)	470-40-6	0.0955	0.1212	0.1433	0.1200	0.0163
(E)-Calamenene	1563(1561)	73209-42-4	0.1816	0.1972	0.2068	0.1952	0.0090
α-Calacorene	1584(1584)	21391-99-1	0.1020	0.0864	0.0943	0.0942	0.0052
**Esters**							
Phenylmethyl acetate	1187(1180)	140-11-4	0.6349	0.8891	0.6745	0.7328	0.1042

Methyl salicylate	1223(1222)	119-36-8	4.2475	3.3183	3.1579	3.5746	0.4486
Txib	1617(1588)	6846-50-0	0.5407	0.4612	0.3667	0.4562	0.0597
**Ketones**							
2-Heptanone	901(891)	110-43-0	0.2303	0.2453	0.2529	0.2428	0.0084
Prenylacetone	1002(995)	110-93-0	1.0826	1.5273	1.2865	1.2988	0.1523
3,5-Octadienone	1089(1074)	38284-27-4	3.9802	4.0705	4.0055	4.0187	0.0345
Jasmone	1427(1415)	488-10-8	1.0659	1.4034	1.0392	1.1695	0.1560
α-Ionone	1456(1438)	127-41-3	0.2072	0.2110	0.2708	0.2297	0.0274
Geranylaceton	1476(1461)	3796-70-1	0.4263	0.4109	0.3827	0.4067	0.0160
2,6-Di-tert-butylquinone	1487(1472)	719-22-2	0.4713	0.4141	0.5441	0.4765	0.0451
(E)-β-Ionone	1500(1490)	79-77-6	2.0657	2.2595	1.7889	2.0380	0.1661
β-Ionone epoxide	1510(1497)	23267-57-4	1.0392	0.9119	0.9877	0.9796	0.0451
**Nitrogen compounds**							
Indole	1328(1329)	120-72-9	1.1258	1.2268	1.0610	1.1379	0.0593
**Oxygen heterocyclic**							
2-Methoxyfuran	806(NA)	25414-22-6	0.9349	0.8819	0.7420	0.8530	0.0740

Table S5. Odor description of aroma components of tea dhools and Chimonanthus tea

Name	CAS	Odor type	Odor description	Reference/source
α-Selinene	473-13-2	Amber	Amber	http://www.perflavory.com/search.php
1,3-Xylene	108-38-3	Aromatic	Aromatic	https://api.chemicalbook.com/
Octane	111-65-9	Fatty	Alkane, Gasoline	https://cosylab.iiitd.edu.in/flavordb2/
(E,E)-2,4-Heptadienal	4314-03-5	Fatty	Fatty	Li et al., 2024a
Penten-3-ol	616-25-1	Fatty	Butter, Fish, Green, Oxidized, Wet Earth	https://www.femaflavor.org/
Phenylmethanol	100-51-6	Floral/sweet	Floral, Rose, Phenolic, Balsamic	Ma et al., 2022
Linalool oxideI	5989-33-3	Floral/sweet	Earthy, Floral, Sweet, Woody	Ma et al., 2022
Linalool oxideII	34995-77-2	Floral/sweet	Floral, Sweet, Grape-Like, Woody	Ma et al., 2022
Linalool	78-70-6	Floral/sweet	Floral, Sweet, Rose	Ma et al., 2022; Chen et al., 2024
Cinnamyl alcohol	104-54-1	Floral/sweet	Floral, Honey, Oil	https://www.femaflavor.org/
Phenylmethanal	100-52-7	Floral/sweet	Floral	Feng et al., 2024
Toluene	108-88-3	Floral/sweet	Paint, Sweet	https://cosylab.iiitd.edu.in/flavordb2/
Limonene	138-86-3	Floral/sweet	Floral, Herb, Sweet	Ma et al., 2022
β-Ocimene	13877-91-3	Floral/sweet	Floral	https://www.femaflavor.org/
Longifolene	475-20-7	Floral/sweet	Rose, Medical, Firneedle, Sweet, Woody	https://cosylab.iiitd.edu.in/flavordb2/
Methyl anthranilate	134-20-3	Floral/sweet	Flower, Honey, Peach	https://www.femaflavor.org/
γ-Phenylpropyl acetate	122-72-5	Floral/sweet	Floral	https://www.femaflavor.org/
(Z)-Cinnamyl acetate	77134-01-1	Floral/sweet	Floral, Sweet, Balsam, Spicy, Cinnamon	https://cosylab.iiitd.edu.in/flavordb2/
(E)-Cinnamyl acetate	103-54-8	Floral/sweet	Floral, Fruit, Honey	https://www.femaflavor.org/
1-Phenylethanone	98-86-2	Floral/sweet	Almonds, Flower, Meat, Must	https://www.femaflavor.org/
Indole	120-72-9	Floral/sweet	Floral, Animal Like	Ma et al., 2022
Geraniol	106-25-2	Floral/sweet	Sweet, Flora	Ma et al., 2022; Chen et al., 2024
Phenylethanal	122-78-1	Floral/sweet	Flowery, Honey-Like	Li et al., 2024a

Decanal	112-31-2	Floral/sweet	Floral, Fried, Orange Peel, Penetrating, Tallow	https://www.femaflavor.org/
Methyl phenylacetate	101-41-7	Floral/sweet	Honey, Jasmine	https://www.femaflavor.org/
Jasmone	488-10-8	Floral/sweet	Floral	https://www.femaflavor.org/
α-Ionone	127-41-3	Floral/sweet	Sweet, Woody, Floral	Ma et al., 2022
Geranylaceton	3796-70-1	Floral/sweet	Rose, Leafy, Floral	Ma et al., 2022
(E)-β-Ionone	79-77-6	Floral/sweet	Floral	Li et al., 2022a
Phenylethanol	60-12-8	Floral/sweet	Sweet, Floral	Ma et al., 2022
Linalool oxide IV	39028-58-5	Floral/sweet	Floral, Woody	Hua et al., 2024
α-Terpieol	10482-56-1	Floral/sweet	Lilac, Floral, Terpenic	http://www.perflavory.com/search.php
Nerolidol	142-50-7	Floral/sweet	Citrus, Waxy, Floral, Woody, Green	https://cosylab.iiitd.edu.in/flavordb2/
(E)-β-Ocimene	3779-61-1	Floral/sweet	Floral	https://www.femaflavor.org/
β-Phenylethyl acetate	103-45-7	Floral/sweet	Flower, Honey, Rose	https://www.femaflavor.org/
Hotrienol	20053-88-7	Floral/sweet	Sweet, Tropical, Ocimene, Fennel, Ginger, Myrcene	http://www.perflavory.com/search.php
β-Damascenone	23726-93-4	Fruity	Apple, Tobacco, Rose, Smoky, Sweet, Honey	https://cosylab.iiitd.edu.in/flavordb2/
2-Methyl-2-pentenal	623-36-9	Fruity	Fruit	https://www.femaflavor.org/
α-Phellandrene	99-83-2	Fruity	Citrus, Fresh, Mint	https://www.femaflavor.org/
α-Terpinene	99-86-5	Fruity	Lemon	https://www.femaflavor.org/
γ-Terpinene	99-85-4	Fruity	Bitter, Citrus	https://www.femaflavor.org/
δ-Cadinene	483-76-1	Fruity	Fruity	https://www.femaflavor.org/
Pentyl acetate	628-63-7	Fruity	Apple, Banana, Pear	https://www.femaflavor.org/
Benzyl formate	104-57-4	Fruity	Fruit	https://www.femaflavor.org/
Phenylmethyl acetate	140-11-4	Fruity	Fruit	https://www.femaflavor.org/
β-Cyclocitral	432-25-7	Fruity	Lemon-Like, Citrus	Li et al., 2024a
Propylacetone	591-78-6	Fruity	Fruity, Fungal, Meaty, Buttery	http://www.perflavory.com/search.php
2-Heptanone	110-43-0	Fruity	Blue Cheese, Fruit	https://www.femaflavor.org/
Prenylacetone	110-93-0	Fruity	Citrus, Mushroom, Pepper, Rubber, Strawberry	https://www.femaflavor.org/

2-Decanone	693-54-9	Fruity	Fat, Fruit	https://www.femaflavor.org/
2-Ethyl-5-methylpyrazine	13360-64-0	Fruity	Fruit, Green	https://www.femaflavor.org/
2,5-Dimethyl-3-isopentylpyrazine	18433-98-2	Fruity	Fruity	http://www.perflavory.com/search.php
2-Heptanol	543-49-7	Fruity	Citrus, Earth, Fried, Mushroom, Oil	https://www.femaflavor.org/
Heptanal	111-71-7	Fruity	Fruity,Cookie	Li et al., 2022a
Octanal	124-13-0	Fruity	Fruity	Feng et al., 2024
Citral	5392-40-5	Fruity	Sweet, Citral, Lemon Peel	Ma et al., 2022； Chen et al., 2024
(E)-Geraniol	106-24-1	Fruity	Geranium, Lemon Peel, Passion Fruit, Peach, Rose	https://www.femaflavor.org/
Dodecanal	112-54-9	Fruity	Fatty, Fruity	Li et al., 2022a
Ethyl Acetate	141-78-6	Fruity	Aromatic, Brandy, Grape	https://www.femaflavor.org/
Propyl acetate	109-60-4	Fruity	Celery, Floral, Pear, Red Fruit	https://www.femaflavor.org/
Pentanol	71-41-0	Fruity	Balsamic, Fruit, Green, Pungent	https://www.femaflavor.org/
Nonanal	124-19-6	Fruity	Citrus-Like, Soapy	Li et al., 2024a
β-Ionone epoxide	23267-57-4	Fruity	Fruit, Wood	https://www.femaflavor.org/
Linalool oxide Pyranoid	14009-71-3	Fruity	Citrus, Green	http://www.perflavory.com/search.php
(Z)-3-Hexenol	928-96-1	Green	Grass, Green Fruit, Green Leaf, Herb, Unripe Banana	https://www.femaflavor.org/
Hexanol	111-27-3	Green	Green, Grassy	Feng et al., 2024
Hexanal	66-25-1	Green	Green, Grassy	Li et al., 2024a
2-Methylpentanal	123-15-9	Green	Green	https://www.femaflavor.org/
(E)-2-Hexanal	6728-26-3	Green	Green, Leaf	Li et al., 2022a
(E)-3-Hexenol	928-97-2	Green	Green, Grassy	Li et al., 2024a; Chen et al., 2024
3,5-Octadienone	38284-27-4	Green	Green, Grassy	Feng et al., 2024
3-Octenol	3391-86-4	Mushroom	Mushroom	Feng et al., 2024
Isobutanal	78-84-2	Nutty	Burnt, Caramel, Cocoa, Green, Malt	https://www.femaflavor.org/
1-(1H-pyrrol-2-yl)ethanone	1072-83-9	Nutty	Bread, Cocoa, Hazelnut, Licorice, Walnut	https://www.femaflavor.org/
2,5-Diethyl pyrazine	13238-84-1	Nutty	Nutty, Hazelnut	http://www.perflavory.com/search.php

3,5-Dimethyl-2-propylpyrazine	32350-16-6	Nutty	Nutty	http://www.perflavory.com/search.php
3-Ethyl-2,5-dimethylpyrazine	13360-65-1	Rice crust	Rice crust	Li et al., 2024a
2,6-Diethyl pyrazine	13067-27-1	Rice crust	Rice crust	Li et al., 2024a
2,3-Diethyl-5-methylpyrazine	18138-04-0	Rice crust	Rice crust	Li et al., 2024a
2,5-Dimethylpyrazine	123-32-0	Roasted	Cocoa, Roast Beef, Roasted Nut	https://www.femaflavor.org/
2-Ethyl-3-methylpyrazine	15707-23-0	Roasted	Green, Must, Nut, Potato, Roasted	https://www.femaflavor.org/
2-Methyl-3,5-diethylpyrazine	18138-05-1	Roasted	Baked, Cocoa, Roast, Rum	https://www.femaflavor.org/
1-Furfurylpyrrole	1438-94-4	Roasted	Cocoa, Green, Roast	https://www.femaflavor.org/
Styrene	100-42-5	Spicy/herbal	Balsamic, Gasoline, Floral, Sweet	https://cosylab.iiitd.edu.in/flavordb2/
3-Methyl-butanal	590-86-3	Spicy/herbal	Malty	Li et al., 2024a
2-Methyl-butanal	96-17-3	Spicy/herbal	Malty	Li et al., 2024a
3-Phenylpropanol	122-97-4	Spicy/herbal	Arise, Cinnamon, Fruit	https://www.femaflavor.org/
(E)-Cinnamaldehyde	14371-10-9	Spicy/herbal	Spice	https://www.femaflavor.org/
Sabinene	3387-41-5	Spicy/herbal	Citrus, Spice, Pepper, Turpentine	https://cosylab.iiitd.edu.in/flavordb2/
Allo-Ocimene	673-84-7	Spicy/herbal	Spicy, Nutty	Ma et al., 2022
δ-EIemene	20307-84-0	Spicy/herbal	Sweet, Herbal, Woody	http://www.perflavory.com/search.php
β-Guaiene	88-84-6	Spicy/herbal	Wood, Spicy, Dry	https://cosylab.iiitd.edu.in/flavordb2/
Methyl salicylate	119-36-8	Spicy/herbal	Peppermint, Sweet, Wintergreen	Ma et al., 2022
Pulegone	15932-80-6	Spicy/herbal	Herbaceous-Minty, Resinous Odor, Pleasant Odor	Göksu Sürücü et al., 2024
Eugenol	97-53-0	Spicy/herbal	Burnt, Clove, Spice	https://www.femaflavor.org/
(E)-Isoeugenol	5932-68-3	Spicy/herbal	Sweet, Spicy, Carnation, Phenolic, Floral	http://www.perflavory.com/search.php
5-Methylfurfural	620-02-0	Spicy/herbal	Spice, Caramel, Maple	http://www.perflavory.com/search.php
3,4-Dehydroionene	30364-38-6	Spicy/herbal	Licorice	https://cosylab.iiitd.edu.in/flavordb2/
2,5-dimethylfuran	625-86-5	Spicy/herbal	Savory	https://www.femaflavor.org/
Pentanal	110-62-3	Spicy/herbal	Almond, Bitter, Malt	https://www.femaflavor.org/
Ethyl salicylate	118-61-6	Spicy/herbal	Spicy, Anisic, Wintergreen-Like Aroma	Göksu Sürücü et al., 2024

Dihydroactinidiolide	17092-92-1	Spicy/herbal	Musk coumarin	http://www.perflavory.com/search.php
Undecanal	112-44-7	Waxy	Waxy, Soapy, Floral, Aldehydic, Citrus	http://www.perflavory.com/search.php
Tetradecanal	124-25-4	Waxy	Waxy, Amber, Incense, Citrus	http://www.perflavory.com/search.php
4-Terpineol	562-74-3	Woody	Earth, Must, Nutmeg, Wood	https://www.femaflavor.org/
α-Thujene	2867-05-2	Woody	Woody, Herb, Green	https://cosylab.iiitd.edu.in/flavordb2/
α-Pinene	80-56-8	Woody	Cedarwood, Pine, Sharp	https://www.femaflavor.org/
β-Pinene	127-91-3	Woody	Pine, Polish, Wood	https://www.femaflavor.org/
β-Myrcene	123-35-3	Woody	Woody	Ma et al., 2022
1,3,8-p-Menthatriene	18368-95-1	Woody	Woody, Herbal	https://cosylab.iiitd.edu.in/flavordb2/
Caryophyllene	87-44-5	Woody	Fried, Spice, Wood	https://www.femaflavor.org/
α-Humulene	6753-98-6	Woody	Woody	https://cosylab.iiitd.edu.in/flavordb2/
α-Muurolene	10208-80-7	Woody	Woody	https://cosylab.iiitd.edu.in/flavordb2/
α-Cedrene	469-61-4	Woody	Woody, Cedar, Sweet	http://www.perflavory.com/search.php
α-Calacorene	21391-99-1	Woody	Woody	http://www.perflavory.com/search.php

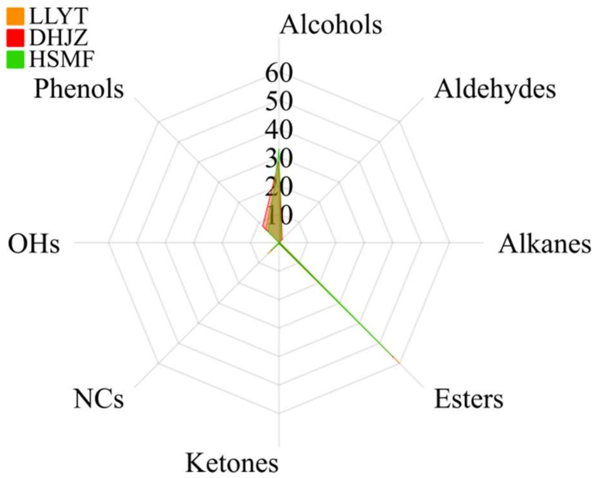

Figure S1. Radar chart of different volatile compounds in three tea dhools after scenting. Abbreviations: LLYT, Large-leaf Yellow Tea; DHJZ, Dianhong jinzhen Tea; HSMFT, Huangshan Maofeng Tea; NCs, Nitrogen compounds; OHs, Oxygen heterocyclics.

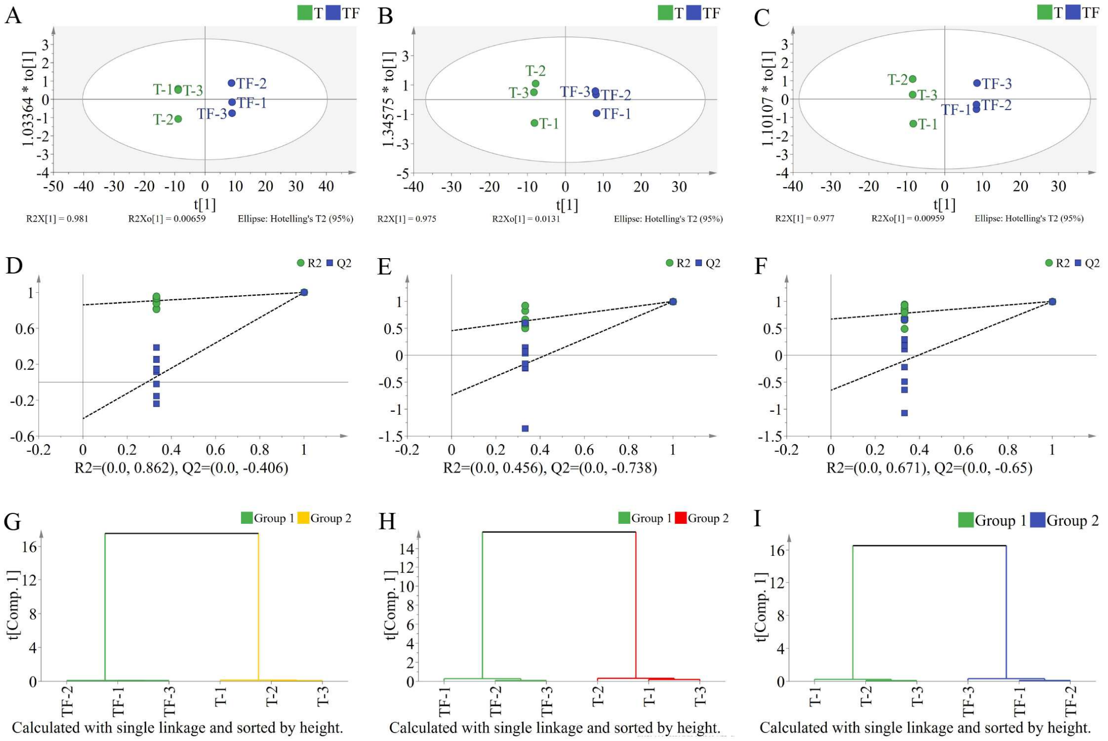

Figure S2. OPLS-DA, permutation test and HCA of three tea dhools before and after scenting. (A) OPLS-DA statistical analysis of volatile components of LLYT. (B) OPLS-DA statistical analysis of volatile components of DHJZ. (C) OPLS-DA statistical analysis of volatile components of HSMF. (D) Cross validation of OPLS-DA model of volatile components of LLYT. (E) Cross validation of OPLS-DA model of volatile components of DHJZ. (F) Cross validation of OPLS-DA model of volatile components of HSMF. (G) Hierarchical cluster analysis (HCA) of volatile components of LLYT. (H) Hierarchical cluster analysis (HCA) of volatile components of DHJZ. (I) Hierarchical cluster analysis (HCA) of volatile components of HSMF. Abbreviations: LLYT, Large-leaf Yellow Tea; DHJZ, Dianhong jinzhen Tea; HSMF, Huangshan Maofeng Tea; T stands for tea without scenting; TF stands for scented tea.

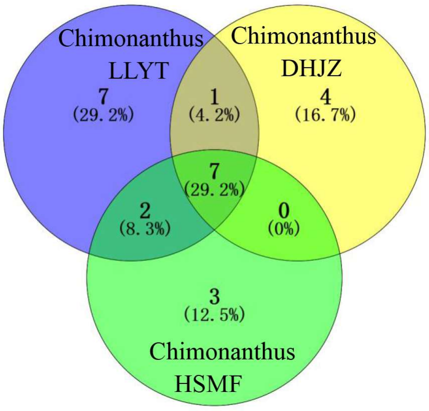

Figure S3. Venn diagram of three kinds of scented tea. Abbreviations: LLYT, Large-leaf Yellow Tea; DHJZ, Dianhong jinzhen Tea; HSMF, Huangshan Maofeng Tea.

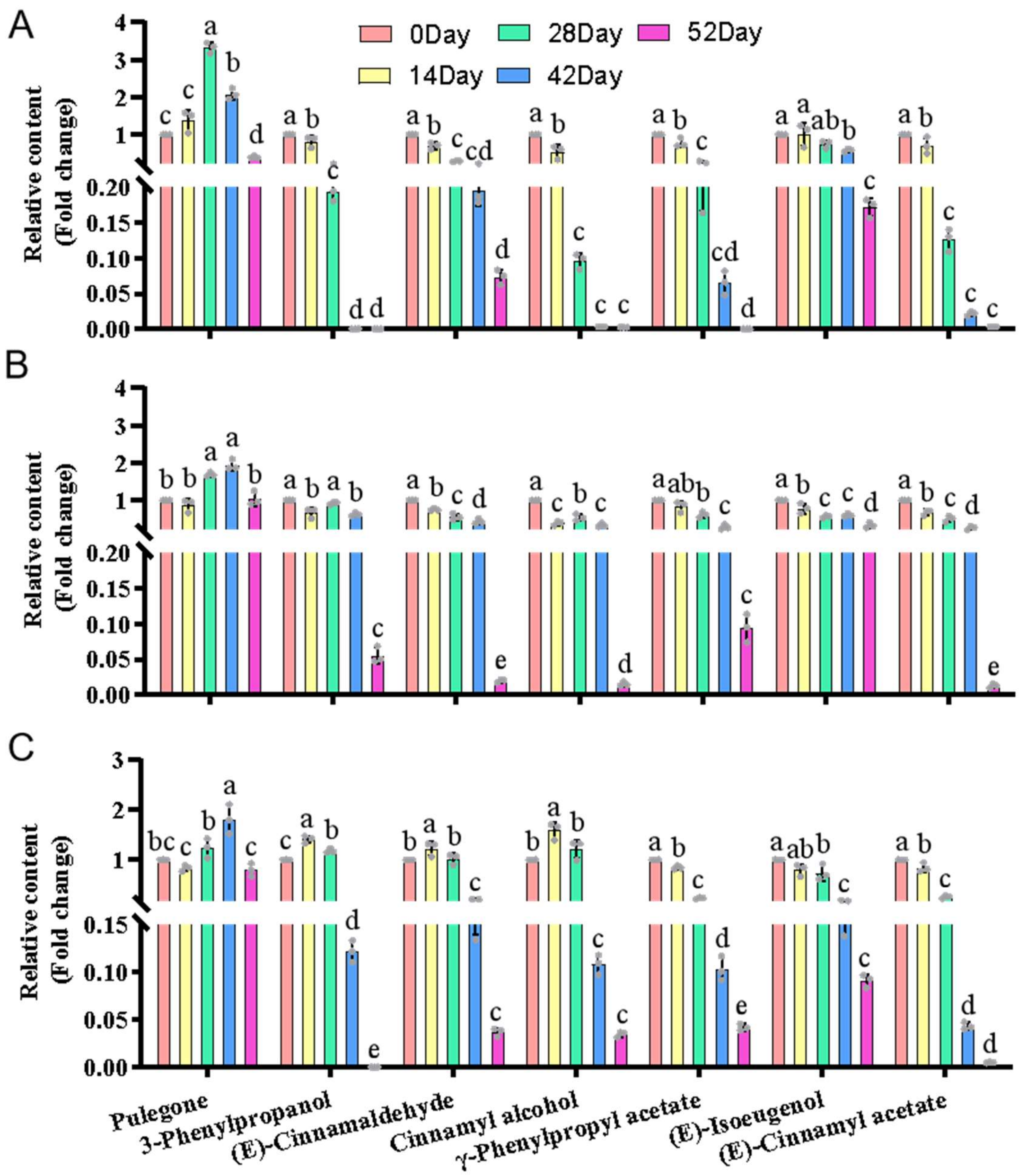

Figure S4. Changes of 7 volatile substances added after scenting during storage. (A) The changes of relative contents of 7 volatile substances in scented LLYT during storage. (B) The changes of relative contents of 7 volatile substances in scented DHJZ during storage. (C) The changes of relative contents of 7 volatile substances in scented HSMF during storage. Data were from three experiments (n = 3) and expressed as means ± s.d., and the significant differences are indicated by letters from one-way ANOVA followed by Tukey's HSD test; the different letters of the same compound indicate significant difference at P <0.05.

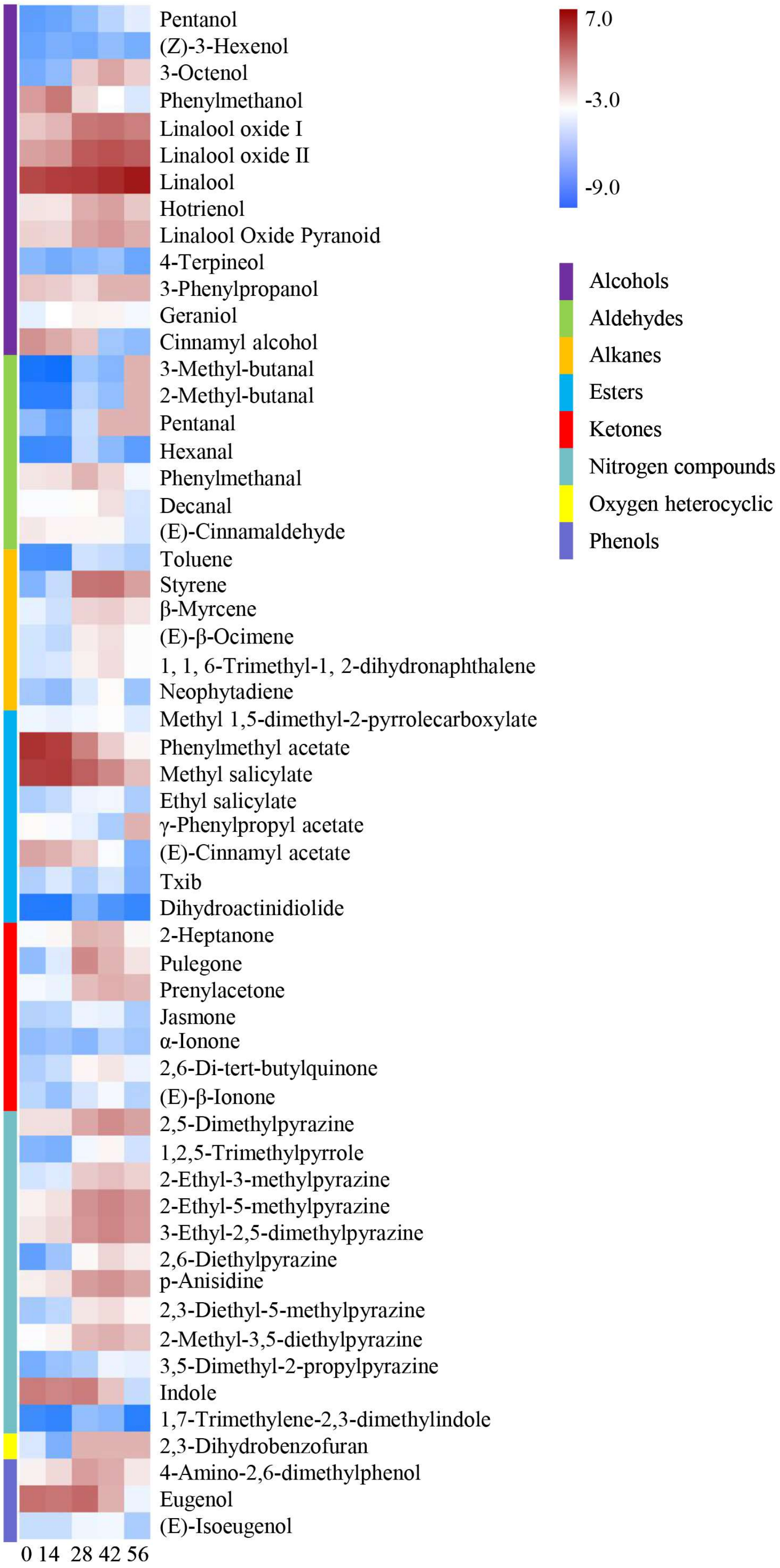

Figure S5. Changes of content percentage of all volatile compounds during storage after scenting of LLYT. Abbreviations: LLYT, Large-leaf Yellow Tea; 0, 14, 28, 42 and 56 represent the number of days of storage.

Table S6. Identification of aroma of Chimonanthus flower-scented LLYT

Name	0		14		28		42		56	
	Precentage of content	SD	Precentage of content	SD	Precentage of content	SD	Precentage of content	SD	Precentage of content	SD
**Alcohols**										
Pentanol	0.0090	0.0001	0.0110	0.0008	0.0192	0.0012	0.0400	0.0034	0.0787	0.0046
(Z)-3-Hexenol	0.0106	0.0004	0.0148	0.0001	0.0124	0.0008	0.0211	0.0023	0.0140	0.0017
3-Octenol	0.0133	0.0006	0.0206	0.0006	0.5440	0.0511	1.4460	0.1269	0.4873	0.0321
Phenylmethanol	1.9331	0.2399	4.9214	0.6829	0.3631	0.0222	0.1287	0.0017	0.0689	0.0023
Linalool oxide I	0.6035	0.0443	0.9434	0.0992	5.2342	0.2384	6.0471	0.5124	4.0718	0.2900
Linalool oxide II	1.6957	0.3119	2.1833	0.0767	10.9966	0.3566	14.0805	1.3223	9.9971	0.9441
Linalool	18.3175	1.3370	25.6731	1.1171	28.3375	1.1391	40.1051	1.6893	69.3550	1.7048
Hotrienol	0.2686	0.0165	0.2657	0.0098	1.1909	0.0333	1.6809	0.0985	0.5783	0.0526
Linalool Oxide IV	0.4343	0.0724	0.3869	0.0346	1.5059	0.1492	2.1434	0.2530	1.1386	0.0256
4-Terpineol	0.0186	0.0014	0.0129	0.0005	0.0190	0.0012	0.0238	0.0008	0.0112	0.0009
3-Phenylpropanol	0.6174	0.0681	0.5026	0.0091	0.3096	0.0292	0.0000	0.0000	0.0000	0.0000
Geraniol	0.0852	0.0077	0.1260	0.0128	0.1925	0.0126	0.1771	0.0123	0.1065	0.0121
Cinnamyl alcohol	2.4684	0.2191	1.2900	0.2154	0.6085	0.0265	0.0275	0.0005	0.0202	0.0016
**Aldehydes**										
3-Methylbutanal	0.0030	0.0004	0.0026	0.0003	0.0266	0.0017	0.0172	0.0023	0.0000	0.0000
2-Methylbutanal	0.0040	0.0003	0.0041	0.0000	0.0383	0.0005	0.0218	0.0008	0.0000	0.0000
Pentanal	0.0205	0.0028	0.0091	0.0003	0.0513	0.0069	0.0000	0.0000	0.0000	0.0000
Hexanal	0.0052	0.0002	0.0056	0.0006	0.0496	0.0038	0.0192	0.0020	0.0088	0.0007
Phenylmethanal	0.2580	0.0126	0.2883	0.0251	0.9698	0.0845	0.3740	0.0492	0.1015	0.0110
Decanal	0.1141	0.0029	0.1142	0.0132	0.1471	0.0102	0.3080	0.0274	0.0649	0.0031

(E)-Cinnamaldehyde	0.2339	0.0056	0.1650	0.0077	0.1706	0.0085	0.1611	0.0043	0.0608	0.0017
**Alkanes**										
Toluene	0.0067	0.0005	0.0063	0.0000	0.0577	0.0037	0.0501	0.0003	0.0341	0.0076
Styrene	0.0167	0.0013	0.0490	0.0032	5.4752	0.8855	5.9338	0.2044	1.6620	0.1528
β-Myrcene	0.0860	0.0015	0.0559	0.0048	0.4149	0.0335	0.4940	0.0278	0.2688	0.0204
(E)-β-Ocimene	0.0613	0.0062	0.0445	0.0044	0.2208	0.0064	0.3145	0.0189	0.1388	0.0133
3,4-Dehydroionene	0.0595	0.0042	0.0677	0.0055	0.1899	0.0372	0.3160	0.0371	0.1379	0.0061
Neophytadiene	0.0284	0.0021	0.0208	0.0009	0.0711	0.0061	0.1444	0.0023	0.0265	0.0024
**Esters**										
Methyl 1,5-dimethy-2-pyrrolecarboxylate	0.0993	0.0137	0.0875	0.0006	0.1030	0.0123	0.1344	0.0126	0.0747	0.0033
Phenylmethyl acetate	34.5011	2.0191	23.7428	0.6273	3.8086	0.1096	0.4986	0.0110	0.1607	0.0200
Methyl salicylate	23.6275	1.1509	26.2999	0.1177	9.4622	0.3593	3.1533	0.3261	0.7694	0.0245
Ethyl salicylate	0.0343	0.0047	0.0480	0.0085	0.0958	0.0090	0.0998	0.0017	0.0332	0.0049
γ-Phenylpropyl acetate	0.1452	0.0164	0.1128	0.0052	0.0825	0.0041	0.0324	0.0004	0.0000	0.0000
(E)-Cinnamyl acetate	1.4871	0.1296	1.0338	0.1002	0.4828	0.0340	0.1142	0.0128	0.0168	0.0007
Txib	0.0355	0.0007	0.0676	0.0085	0.0333	0.0007	0.0646	0.0025	0.0150	0.0003
Dihydroactinidiolide	0.0419	0.0055	0.0231	0.0032	0.0664	0.0063	0.1067	0.0082	0.0380	0.0049
**Ketones**										
2-Heptanone	0.0036	0.0001	0.0035	0.0003	0.0179	0.0005	0.0072	0.0003	0.0048	0.0005
Pulegone	0.1133	0.0131	0.1556	0.0012	0.9687	0.0405	0.8355	0.1035	0.1565	0.0068
Prenylacetone	0.0211	0.0019	0.0726	0.0035	3.0978	0.0734	0.9763	0.0444	0.2720	0.0046
Jasmone	0.1078	0.0085	0.0913	0.0051	0.7799	0.0236	1.1108	0.0609	0.8618	0.0210
α-Ionone	0.0377	0.0041	0.0419	0.0018	0.0961	0.0036	0.0865	0.0021	0.0321	0.0013
2,6-Di-tert-butylquinone	0.0210	0.0009	0.0258	0.0029	0.0180	0.0012	0.0405	0.0015	0.0275	0.0014
(E)-β-Ionone	0.0347	0.0002	0.0525	0.0054	0.1644	0.0196	0.2530	0.0311	0.0921	0.0090

**Nitrogen compounds**										
2,5-Dimethylpyrazine	0.3011	0.0220	0.3075	0.0141	1.3826	0.0989	2.9257	0.2452	1.5692	0.2291
1,2,5-Trimethylpyrrole	0.0169	0.0007	0.0145	0.0005	0.1000	0.0081	0.1665	0.0299	0.0583	0.0017
2-Ethyl-3-methylpyrazine	0.0604	0.0063	0.0727	0.0038	0.5346	0.0254	0.7173	0.0809	0.4457	0.0551
2-Ethyl-5-methylpyrazine	0.1916	0.0094	0.2872	0.0278	2.3841	0.0468	3.5487	0.2147	2.0044	0.2525
3-Ethyl-2,5-dimethylpyrazine	0.2490	0.0272	0.3673	0.0156	2.2478	0.0387	3.5147	0.1633	1.9809	0.1103
2,6-Diethylpyrazine	0.0098	0.0017	0.0261	0.0024	0.1548	0.0096	0.4011	0.0360	0.2161	0.0104
p-Anisidine	0.2036	0.0176	0.3066	0.0269	1.7975	0.1144	2.4784	0.1015	1.4127	0.0623
2,3-Diethyl-5-methylpyrazine	0.0291	0.0015	0.0430	0.0028	0.2498	0.0268	0.3491	0.0311	0.1673	0.0067
2-Methyl-3,5-diethylpyrazine	0.1225	0.0037	0.1773	0.0043	0.8445	0.0521	1.0896	0.1478	0.6523	0.0209
3,5-Dimethyl-2-propylpyrazine	0.0139	0.0010	0.0242	0.0040	0.0367	0.0012	0.0960	0.0162	0.0844	0.0060
Indole	4.4481	0.4152	3.4637	0.0224	4.3614	0.4504	0.6463	0.0174	0.0503	0.0018
1,7-Trimethylene-2,3-dimethylindole	0.0055	0.0006	0.0045	0.0001	0.0219	0.0006	0.0180	0.0002	0.0039	0.0003
**Oxygen heterocyclic**										
2,3-Dihydrobenzofuran	0.0683	0.0068	0.0152	0.0001	0.0000	0.0000	0.0000	0.0000	0.0000	0.0000
**Phenols**										
4-Amino-2,6-dimethylphenol	0.1878	0.0175	0.3559	0.0292	1.7583	0.1152	1.2572	0.1101	0.2420	0.0054
Eugenol	6.3573	0.5829	5.4392	0.2707	7.5157	0.2162	1.0984	0.1026	0.0946	0.0088
(E)-Isoeugenol	0.0515	0.0036	0.0516	0.0067	0.0986	0.0138	0.1040	0.0061	0.0315	0.0023

Abbreviations are as follows: LLYT, Large-leaf Yellow Tea; 0, 14, 28, 42 and 56 represent the number of days of storage.

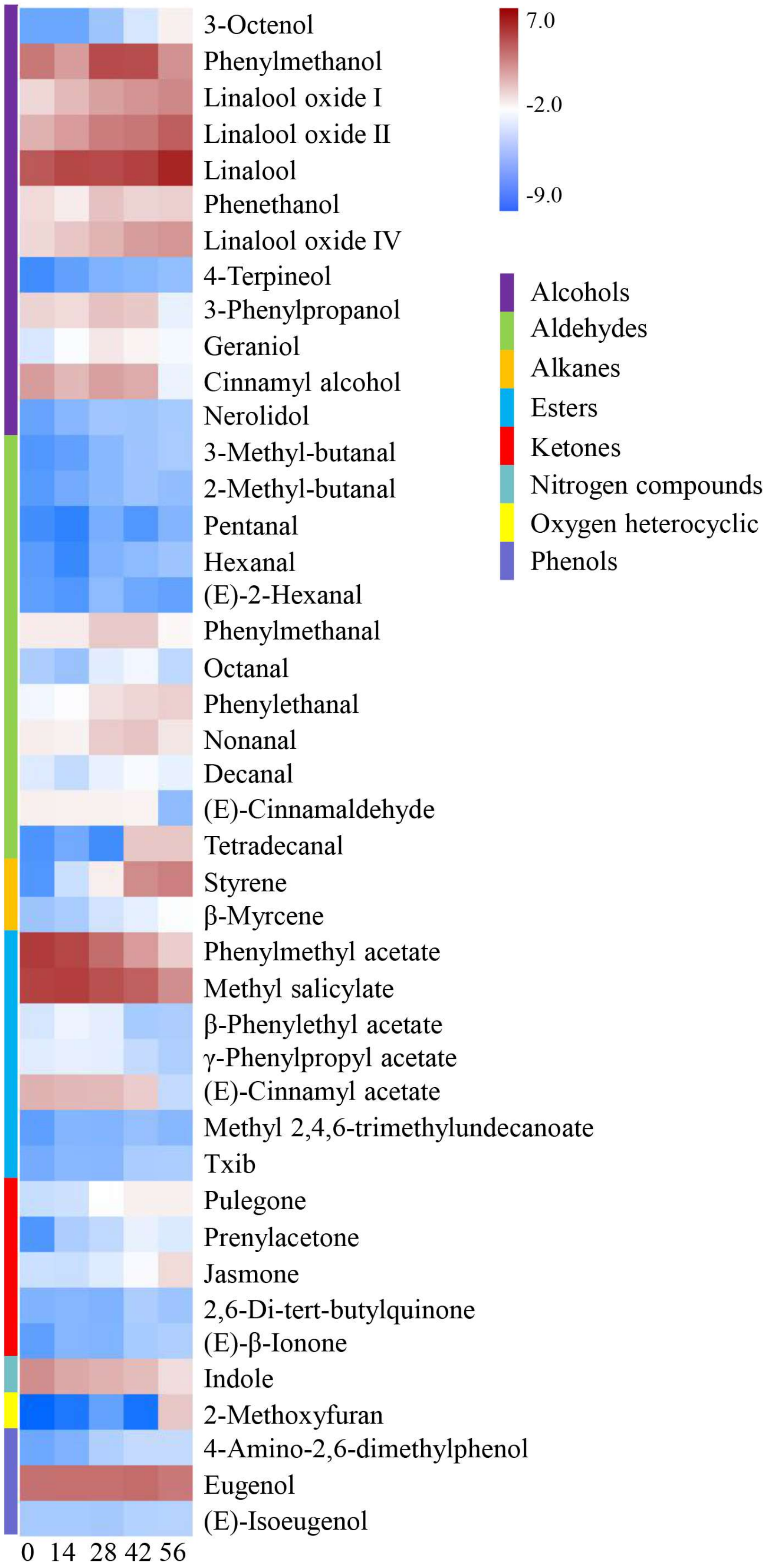

Figure S6. Changes of content percentage of all volatile compounds during storage after scenting of DHJZ. Abbreviations: DHJZ, Dianhong jinzhen Tea; 0, 14, 28, 42 and 56 represent the number of days of storage.

Table S7. Identification of aroma of Chimonanthus flower-scented DHJZ

Name	0		14		28		42		56	
	Precentage of content	SD	Precentage of content	SD	Precentage of content	SD	Precentage of content	SD	Precentage of content	SD
**Alcohols**										
3-Octenol	0.0152	0.0007	0.0155	0.0007	0.0403	0.0043	0.1153	0.0122	0.3765	0.0290
Phenylmethanol	6.8858	0.4043	2.8626	0.1229	20.5716	1.7349	19.4897	1.5624	3.6798	0.4534
Linalool oxide I	0.6759	0.0180	1.4178	0.2856	2.6003	0.1464	3.4568	0.2987	4.6399	0.4373
Linalool oxide II	1.7085	0.0247	2.9118	0.0914	6.1005	0.3470	7.0844	0.3203	12.9025	1.0956
Linalool	14.5692	1.0194	22.7459	2.0139	20.5041	0.6126	26.6394	0.4428	51.8455	2.2348
Phenylethanol	0.6320	0.0328	0.4098	0.0194	1.1341	0.6520	0.7183	0.1183	0.8008	0.0519
Linalool oxide IV	0.6635	0.0190	1.0319	0.1501	1.5925	0.1355	2.8206	0.0811	3.3264	0.0463
4-Terpineol	0.0067	0.0003	0.0127	0.0022	0.0221	0.0009	0.0259	0.0003	0.0326	0.0027
3-Phenylpropanol	0.7303	0.0484	0.6206	0.0390	1.1410	0.0078	0.9759	0.0111	0.1662	0.0174
Geraniol	0.1239	0.0094	0.2359	0.0233	0.4567	0.0328	0.3410	0.0297	0.2039	0.0305
Cinnamyl alcohol	2.8575	0.2998	1.4550	0.1866	2.6038	0.3635	1.9595	0.0687	0.1746	0.0136
Nerolidol	0.0141	0.0014	0.0262	0.0033	0.0425	0.0079	0.0408	0.0018	0.0477	0.0037
**Aldehydes**										
3-Methylbutanal	0.0094	0.0005	0.0127	0.0031	0.0276	0.0020	0.0398	0.0004	0.0494	0.0023
2-Methylbutanal	0.0105	0.0004	0.0180	0.0030	0.0275	0.0019	0.0389	0.0029	0.0317	0.0036
Pentanal	0.0071	0.0004	0.0049	0.0005	0.0192	0.0012	0.0093	0.0010	0.0234	0.0008
Hexanal	0.0110	0.0019	0.0060	0.0002	0.0225	0.0014	0.0293	0.0014	0.0402	0.0019
(E)-2-Hexanal	0.0119	0.0017	0.0092	0.0007	0.0293	0.0027	0.0159	0.0004	0.0132	0.0012
Phenylmethanal	0.4117	0.0267	0.4120	0.0129	0.9351	0.0260	0.9311	0.0683	0.2985	0.0232
Octanal	0.0528	0.0038	0.0365	0.0024	0.1446	0.0121	0.1968	0.0084	0.0736	0.0068

Phenylethanal	0.1937	0.0164	0.2453	0.0077	0.5353	0.0321	0.6888	0.0293	0.8269	0.0560
Nonanal	0.3970	0.0584	0.3438	0.0202	0.9107	0.0858	1.0628	0.1389	0.4632	0.0230
Decanal	0.1323	0.0221	0.0807	0.0048	0.1627	0.0251	0.2155	0.0135	0.1642	0.0172
(E)-Cinnamaldehyde	0.3744	0.0398	0.3690	0.0360	0.3465	0.0175	0.3414	0.0210	0.0302	0.0021
Tetradecanal	0.0085	0.0009	0.0173	0.0047	0.0071	0.0001	0.0000	0.0000	0.0000	0.0000
**Alkanes**										
Styrene	0.0092	0.0015	0.0942	0.0061	0.3818	0.0230	4.3544	0.5954	5.8513	0.6503
β-Myrcene	0.0403	0.0031	0.0515	0.0036	0.1049	0.0120	0.1551	0.0036	0.2400	0.0305
**Esters**										
Phenylmethyl acetate	30.6627	1.5508	23.4252	1.3176	9.0991	0.6327	2.9221	0.3964	0.8504	0.0828
Methyl salicylate	24.4872	0.9176	28.2554	1.4571	17.9692	3.0221	12.7003	0.9679	3.9869	0.3735
β-Phenylethyl acetate	0.1156	0.0163	0.1826	0.0038	0.1523	0.0171	0.0472	0.0051	0.0535	0.0028
γ-Phenylpropyl acetate	0.1430	0.0144	0.1549	0.0155	0.1486	0.0145	0.0820	0.0013	0.0553	0.0049
(E)-Cinnamyl acetate	1.6148	0.1126	1.4210	0.0893	1.3659	0.1065	0.8734	0.0447	0.0823	0.0040
Methyl 2,4,6-trimethylundecanoate	0.0120	0.0008	0.0245	0.0008	0.0238	0.0016	0.0334	0.0012	0.0256	0.0021
Txib	0.0183	0.0007	0.0267	0.0058	0.0263	0.0062	0.0512	0.0013	0.0518	0.0018
**Ketones**										
Pulegone	0.0892	0.0054	0.0999	0.0031	0.2569	0.0191	0.3704	0.0317	0.3785	0.0101
Prenylacetone	0.0090	0.0006	0.0531	0.0484	0.0774	0.0053	0.1649	0.0113	0.1304	0.0028
Jasmone	0.0950	0.0056	0.0920	0.0035	0.1374	0.0125	0.2222	0.0214	0.6254	0.0749
2,6-Di-tert-butylquinone	0.0227	0.0012	0.0246	0.0014	0.0225	0.0019	0.0532	0.0029	0.0395	0.0032
(E)-β-Ionone	0.0120	0.0002	0.0249	0.0021	0.0230	0.0007	0.0469	0.0072	0.0552	0.0010
**Nitrogen compounds**										
Indole	4.0046	0.1081	2.1306	0.4014	1.7110	0.0866	1.3160	0.1791	0.5857	0.0707
**Oxygen heterocyclic**										

2-Methoxyfuran	0.0021	0.0003	0.0035	0.0002	0.0132	0.0016	0.0030	0.0003	0.0000	0.0000
**Phenols**										
4-Amino-2,6-dimethylphenol	0.0159	0.0023	0.0219	0.0012	0.0575	0.0051	0.0819	0.0078	0.0824	0.0077
Eugenol	8.0961	0.0631	8.5642	0.7273	8.4062	0.3183	9.2289	0.7591	6.6323	0.2012
(E)-Isoeugenol	0.0475	0.0019	0.0484	0.0062	0.0455	0.0050	0.0610	0.0039	0.0626	0.0021

Abbreviations are as follows: DHJZ, Dianhong jinzhen Tea; 0, 14, 28, 42 and 56 represent the number of days of storage.

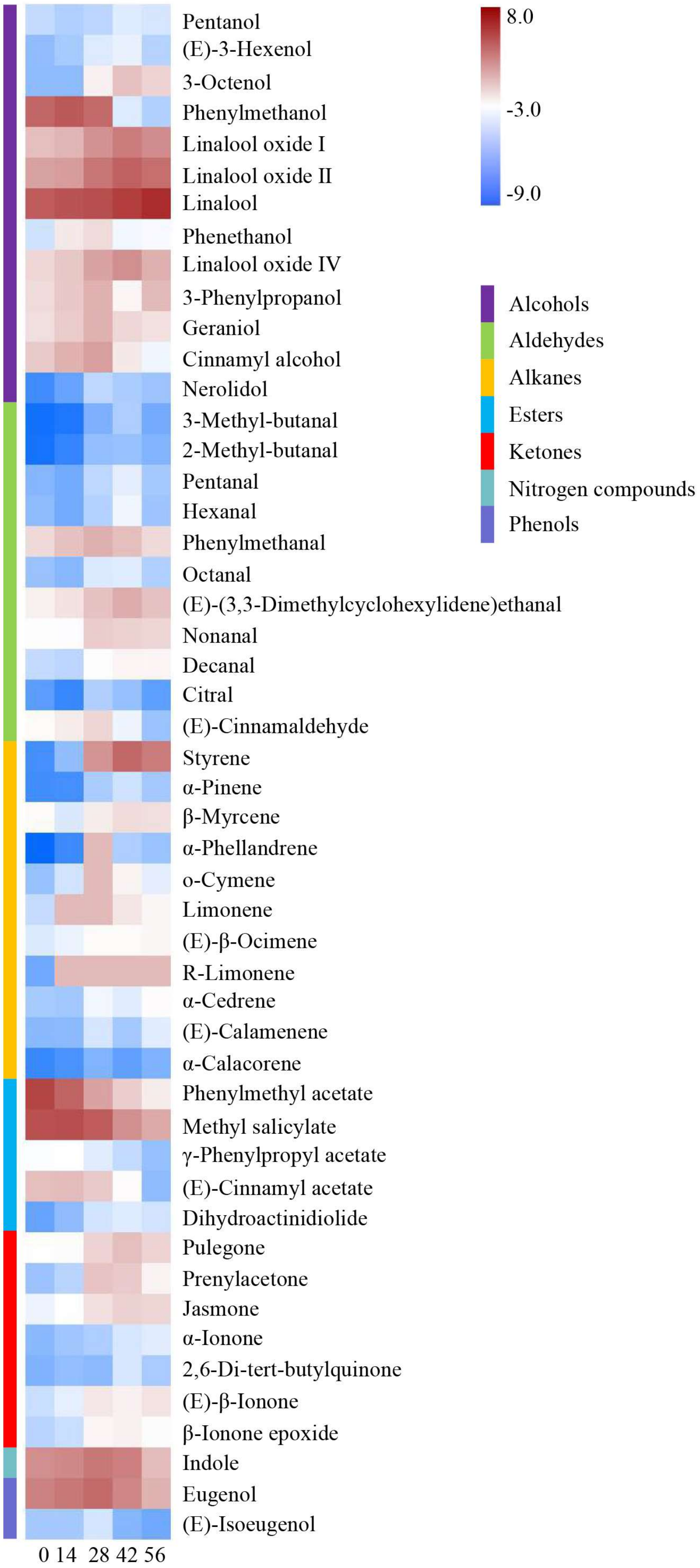

Figure S7. Changes of content percentage of all volatile compounds during storage after scenting of HSMF. Abbreviations: HSMF, Huangshan Maofeng Tea; 0, 14, 28, 42 and 56 represent the number of days of storage.

Table S8. Identification of aroma of Chimonanthus flower-scented HSMF

Name	0		14		28		42		56	
	Precentage of content	SD	Precentage of content	SD	Precentage of content	SD	Precentage of content	SD	Precentage of content	SD
**Alcohols**										
Pentanol	0.0483	0.0009	0.0360	0.0029	0.0422	0.0031	0.0742	0.0039	0.0650	0.0045
(E)-3-Hexenol	0.0211	0.0011	0.0306	0.0040	0.0729	0.0012	0.0882	0.0032	0.0382	0.0018
3-Octenol	0.0202	0.0028	0.0204	0.0012	0.1921	0.0060	0.8069	0.0335	0.4707	0.0631
Phenylmethanol	11.8087	1.8111	18.3354	1.2172	10.9188	1.6348	0.0732	0.0038	0.0365	0.0056
Linalool oxide I	0.8858	0.0590	1.1598	0.1534	3.1840	0.2924	6.2708	0.6668	3.7958	0.2935
Linalool oxide II	2.0425	0.0652	2.4375	0.1052	7.7122	0.5898	13.8290	1.6040	9.5381	0.6893
Linalool	16.3674	1.0206	21.6096	1.8841	24.1304	1.6748	40.5118	1.4438	68.9519	0.8668
Phenethanol	0.0575	0.0057	0.2529	0.0063	0.3744	0.0212	0.1013	0.0122	0.1128	0.0126
Linalool oxide IV	0.4150	0.0170	0.6676	0.0442	2.0118	0.1045	3.6364	0.0873	1.3358	0.1916
3-Phenylpropanol	0.3698	0.0025	0.6575	0.0079	1.2883	0.0733	0.1732	0.0246	0.0000	0.0000
Geraniol	0.3412	0.0296	0.5904	0.0755	1.2781	0.0617	0.4212	0.0591	0.3132	0.0058
Cinnamyl alcohol	0.6224	0.0405	1.2575	0.1880	2.2101	0.1782	0.2554	0.0040	0.0985	0.0012
Nerolidol	0.0057	0.0007	0.0108	0.0001	0.0428	0.0053	0.0313	0.0010	0.0254	0.0006
**Aldehydes**										
3-Methylbutanal	0.0027	0.0003	0.0032	0.0003	0.0152	0.0003	0.0338	0.0018	0.0130	0.0016
2-Methylbutanal	0.0028	0.0003	0.0046	0.0001	0.0218	0.0013	0.0234	0.0023	0.0168	0.0010
Pentanal	0.0176	0.0010	0.0128	0.0006	0.0431	0.0040	0.0816	0.0020	0.0294	0.0026
Hexanal	0.0203	0.0002	0.0129	0.0003	0.0371	0.0008	0.0991	0.0075	0.0258	0.0021
Phenylmethanal	0.3928	0.0169	0.7856	0.0225	1.3477	0.0598	0.8879	0.0233	0.3911	0.0280
Octanal	0.0243	0.0016	0.0185	0.0020	0.0712	0.0002	0.0764	0.0063	0.0352	0.0036

(E)-(3,3-Dimethyl-cyclohexylidene)ethanal	0.1961	0.0129	0.3058	0.0304	0.7940	0.0898	1.5165	0.1326	0.8197	0.0493
Nonanal	0.1360	0.0009	0.1222	0.0131	0.5888	0.0471	0.4986	0.0436	0.4339	0.0172
Decanal	0.0485	0.0022	0.0425	0.0047	0.1333	0.0045	0.1751	0.0197	0.1704	0.0044
Citral	0.0088	0.0004	0.0050	0.0000	0.0345	0.0032	0.0227	0.0012	0.0093	0.0014
(E)-Cinnamaldehyde	0.1459	0.0171	0.2237	0.0199	0.4330	0.0340	0.0967	0.0164	0.0245	0.0028
**Alkanes**										
Styrene	0.0061	0.0003	0.0213	0.0014	3.0817	0.0984	11.7288	0.9072	6.4480	0.0949
α-Pinene	0.0059	0.0003	0.0061	0.0004	0.0316	0.0015	0.0578	0.0036	0.0286	0.0033
β-Myrcene	0.1460	0.0168	0.0697	0.0008	0.2238	0.0196	0.3617	0.0448	0.3359	0.0206
α-Phellandrene	0.0022	0.0000	0.0053	0.0007	0.0000	0.0000	0.0334	0.0039	0.0248	0.0021
o-Cymene	0.0236	0.0019	0.0600	0.0058	0.0000	0.0000	0.1836	0.0207	0.0812	0.0057
Limonene	0.0500	0.0040	0.0000	0.0000	0.0000	0.0000	0.2712	0.0178	0.1660	0.0211
(E)-β-Ocimene	0.0717	0.0044	0.0907	0.0010	0.1495	0.0112	0.1489	0.0006	0.1662	0.0140
R-Limonene	0.0122	0.0001	0.0000	0.0000	0.0000	0.0000	0.0000	0.0000	0.0000	0.0000
α-Cedrene	0.0301	0.0037	0.0281	0.0003	0.1013	0.0016	0.0778	0.0051	0.1448	0.0116
(E)-Calamenene	0.0187	0.0012	0.0197	0.0044	0.0649	0.0106	0.0288	0.0026	0.0787	0.0006
α-Calacorene	0.0051	0.0006	0.0067	0.0004	0.0161	0.0019	0.0098	0.0003	0.0160	0.0001
**Esters**										
Phenylmethyl acetate	32.5599	0.6431	13.1866	0.6476	1.9648	0.0873	0.5733	0.0111	0.2212	0.0088
Methyl salicylate	22.7204	1.0111	24.6633	1.8520	16.3785	1.2247	3.4651	0.3165	1.6239	0.0779
γ-Phenylpropyl acetate	0.1205	0.0048	0.1278	0.0020	0.0773	0.0010	0.0473	0.0045	0.0231	0.0006
(E)-Cinnamyl acetate	0.8748	0.0158	0.9382	0.1222	0.6267	0.0637	0.1442	0.0130	0.0204	0.0002
Dihydroactinidiolide	0.0105	0.0003	0.0207	0.0018	0.0607	0.0038	0.0752	0.0009	0.0598	0.0062
**Ketones**										
Pulegone	0.1311	0.0075	0.1372	0.0042	0.4755	0.0447	0.8991	0.0830	0.4737	0.0314

Prenylacetone	0.0249	0.0012	0.0404	0.0029	0.7641	0.0678	0.6294	0.0591	0.1827	0.0159
Jasmone	0.0961	0.0055	0.1242	0.0068	0.3272	0.0251	0.5010	0.0094	0.4378	0.0205
α-Ionone	0.0188	0.0011	0.0271	0.0013	0.0334	0.0035	0.0649	0.0041	0.0778	0.0067
2,6-Di-tert-butylquinone	0.0160	0.0019	0.0216	0.0033	0.0195	0.0008	0.0652	0.0021	0.0309	0.0012
(E)-β-Ionone	0.0530	0.0042	0.0823	0.0042	0.2629	0.0232	0.1923	0.0009	0.2933	0.0288
β-Ionone epoxide	0.0399	0.0029	0.0534	0.0018	0.1714	0.0070	0.1952	0.0213	0.1379	0.0024
**Nitrogen compounds**										
Indole	3.6406	0.1907	4.3619	0.5462	7.1906	0.3747	5.7199	0.4676	0.9473	0.0463
**Phenols**										
Eugenol	5.2908	0.1058	7.2758	0.1534	10.9380	0.4109	4.7237	0.0996	1.2169	0.0149
(E)-Isoeugenol	0.0293	0.0029	0.0293	0.0030	0.0630	0.0067	0.0176	0.0005	0.0122	0.0014

Abbreviations are as follows: HSMF, Huangshan Maofeng Tea; 0, 14, 28, 42 and 56 represent the number of days of storage.

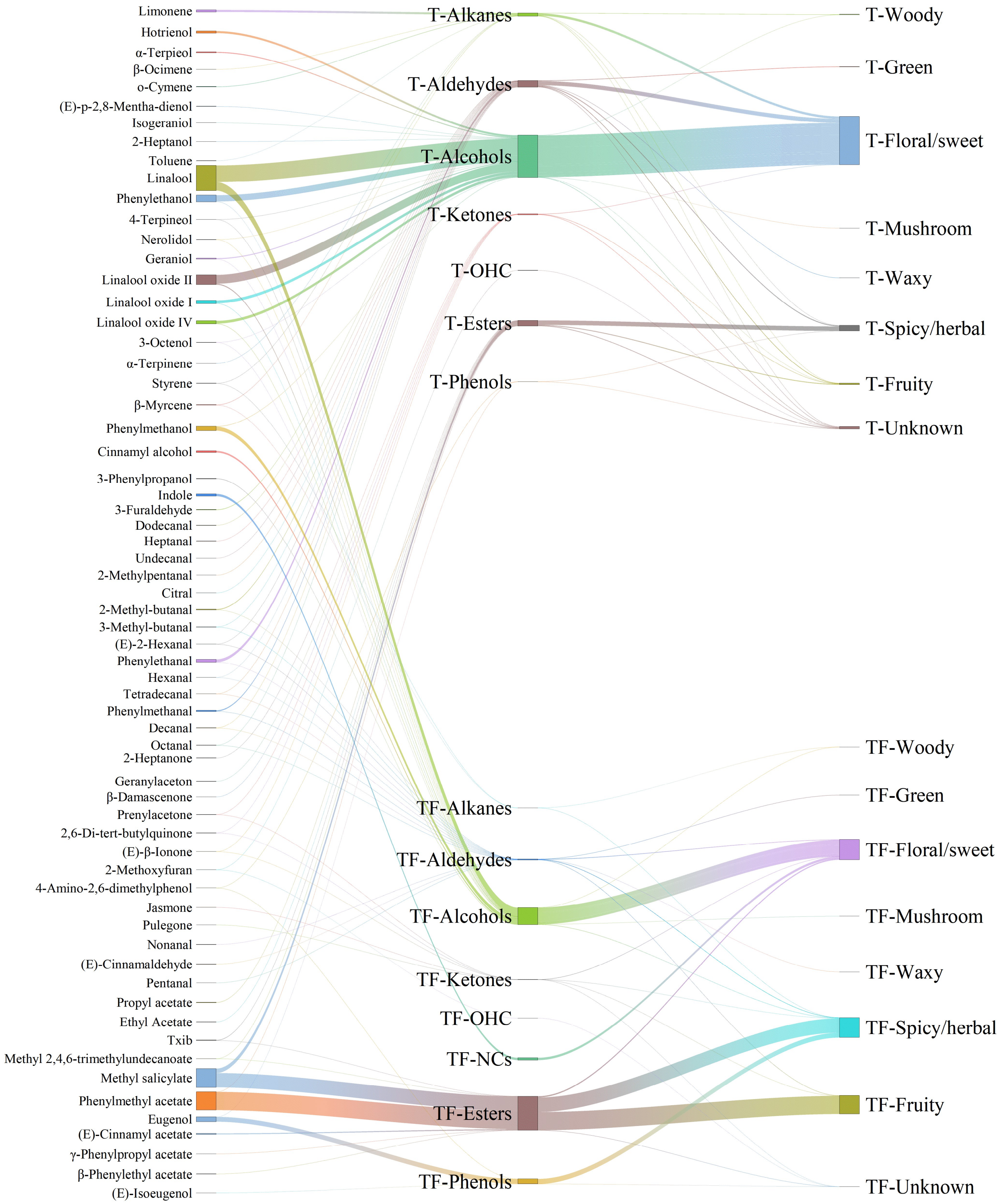

Figure S8. DHJZ dhool vs scented DHJZ flavor sankey diagram. The bars on the left represent volatile substances that contribute to the flavor; the right represents the flavor characteristics; the middle bar represents the categories of aroma volatiles in tea and scented tea; the width of a line indicates the percentage content of the corresponding volatile substance. Abbreviations: T stands for tea without scenting; TF stands for scented tea, NCs, Nitrogen compounds, OHC, Oxygen heterocyclic.

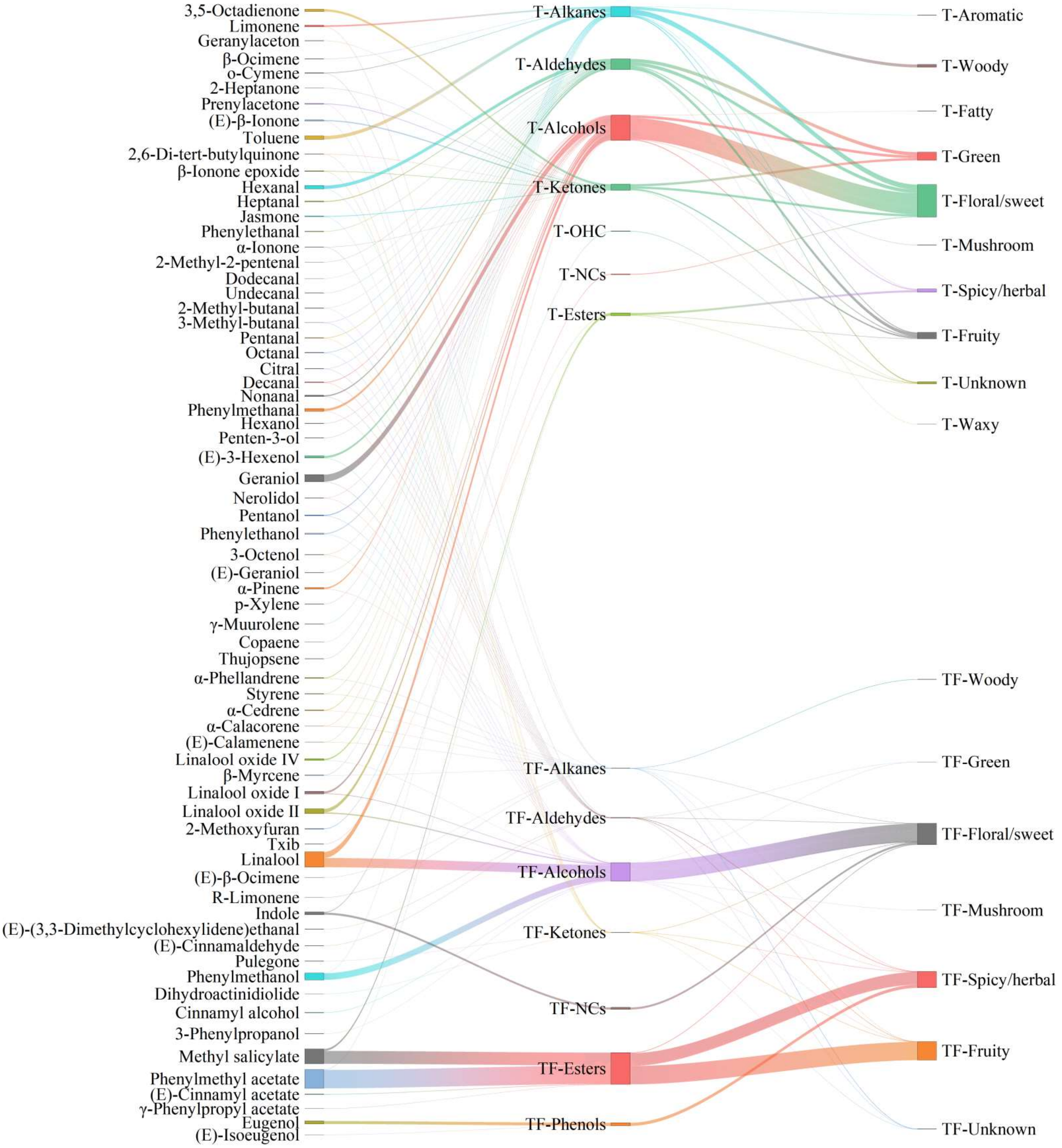

Figure S9. HSMF dhool vs scented HSMF flavor sankey diagram. The bars on the left represent volatile substances that contribute to the flavor; the right represents the flavor characteristics; the middle bar represents the categories of aroma volatiles in tea and scented tea; the width of a line indicates the percentage content of the corresponding volatile substance. Abbreviations: T stands for tea without scenting; TF stands for scented tea, NCs, Nitrogen compounds, OHC, Oxygen heterocyclic.
